# Gene model for the ortholog *Myc* in *Drosophila ananassae*

**DOI:** 10.17912/micropub.biology.000856

**Published:** 2024-11-30

**Authors:** Abigail Myers, Alexa Hoffman, Mindy Natysin, Andrew M Arsham, Joyce Stamm, Jeffrey S. Thompson, Chinmay P. Rele, Laura K Reed

**Affiliations:** 1 University of Alabama, Tuscaloosa, Alabama, US; 2 University of Evansville, Evansville, Indiana, US; 3 Bemidji State University, Bemidji, Minnesota, US; 4 Denison University, Granville, Ohio, US; 5 The University of Alabama, Tuscaloosa, AL USA; 6 Biological Sciences, University of Alabama, Tuscaloosa, Alabama, US

## Abstract

Gene model for the ortholog of Myc ( *Myc* ) in the May 2011 (Agencourt dana_caf1/DanaCAF1) Genome Assembly (GenBank Accession: GCA_000005115.1 ) of *Drosophila ananassae* . This ortholog was characterized as part of a developing dataset to study the evolution of the Insulin/insulin-like growth factor signaling pathway (IIS) across the genus *Drosophila* using the Genomics Education Partnership gene annotation protocol for Course-based Undergraduate Research Experiences.

**Figure 1. Genomic neighborhood and gene model for Myc in Drosophila ananassae:**
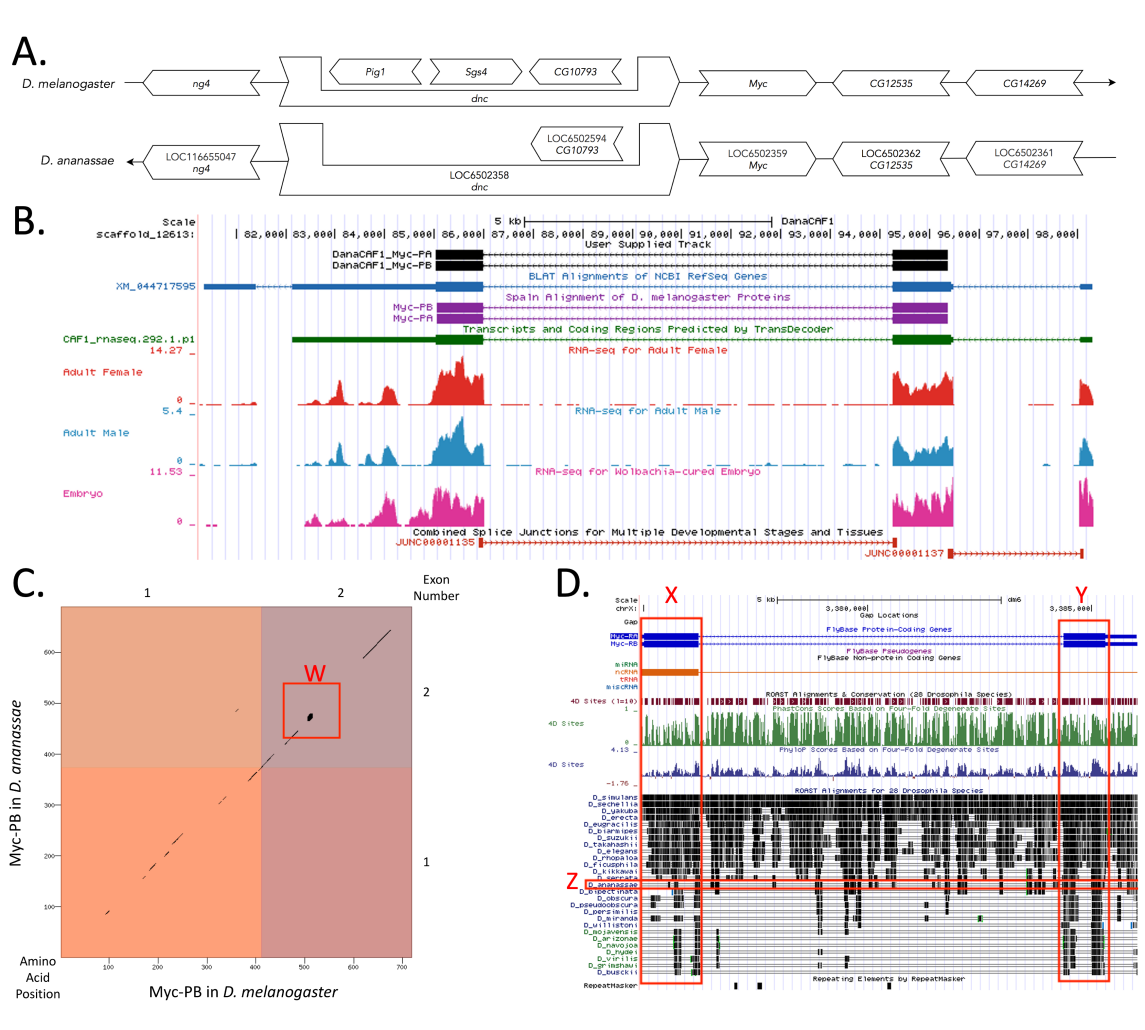
**
(A) Synteny comparison of the genomic neighborhoods for *Myc* in *Drosophila melanogaster* and *D.* *ananassae* .** Thin underlying arrows indicate the DNA strand within which the target gene– *Myc* –is located in *D. melanogaster* (top) and *D. ananassae* (bottom). Thin arrow(s) pointing to the right indicate(s) that *Myc* is on the positive (+) strand in *D. ananassae* and *D.melanogaster* . The wide gene arrows pointing in the same direction as *Myc* are on the same strand relative to the thin underlying arrows, while wide gene arrows pointing in the opposite direction of *Myc* are on the opposite strand relative to the thin underlying arrows. White gene arrows in *D. ananassae* indicate orthology to the corresponding gene in *D. melanogaster* . Gene symbols given in the *D. ananassae* gene arrows indicate the orthologous gene in *D. melanogaster* , while the locus identifiers are specific to *D. ananassae* . **(B) Gene Model in GEP UCSC Track Data Hub (Raney et al. 2014).** The coding-regions of *Myc* in *D. ananassae* are displayed in the User Supplied Track (black); CDSs are depicted by thick rectangles and introns by thin lines with arrows indicating the direction of transcription. Subsequent evidence tracks include BLAT Alignments of NCBI RefSeq Genes (dark blue, alignment of Ref-Seq genes for *D. ananassae* ), Spaln of *D. melanogaster* Proteins (purple, alignment of Ref-Seq proteins from *D. melanogaster* ), Transcripts and Coding Regions Predicted by TransDecoder (dark green), RNA-Seq from Adult Females, Adult Males, and *Wolbachia* -cured Embryos (red, light blue, and pink respectively; alignment of Illumina RNA-Seq reads from *D. ananassae* ), and Splice Junctions Predicted by regtools using *D. ananassae* RNA-Seq (Graveley *et al.* , 2011; SRP006203 , SRP007906 ; PRJNA257286 , PRJNA388952 ). Splice junctions shown have a read-depth of >1000 supporting reads in red. **(C) Dot Plot of Myc-PB in *D. melanogaster* ( *x* -axis) vs. the orthologous peptide in *D. ananassae* ( *y* -axis).** Amino acid number is indicated along the left and bottom; CDS number is indicated along the top and right, and CDSs are also highlighted with alternating colors. Tandem repeats of serine are present in both sequences of the second CDS represented by the red box, Box W. (D) The Conservation Track of 28 *Drosophila* Species compared to CDSs one and two of *D. melanogaster* Myc-RA and Myc-RB contains many regions having lack of sequence similarity (vertical red boxes, Box X and Y; *D. ananassae* is highlighted in the horizontal red box, Box Z).

## Description

**Table:** 

*This article reports a predicted gene model generated by undergraduate work using a structured gene model annotation protocol defined by the Genomics Education Partnership (GEP; thegep.org) for Course-based Undergraduate Research Experience (CURE). The following information may be repeated in other articles submitted by participants using the same GEP CURE protocol for annotating Drosophila species orthologs of D. melanogaster genes in the insulin signaling pathway (ISP).* In this GEP CURE protocol students use web-based tools to manually annotate genes in non-model *Drosophila* species based on orthology to genes in the well-annotated model organism fruitfly *Drosophila melanogaster* (Rele et al., 2023) . Computational-based gene predictions in any organism are often improved by careful manual annotation and curation, allowing for more accurate analyses of gene and genome evolution (Mudge and Harrow 2016; Tello-Ruiz et al., 2019) . These models of orthologous genes across species, such as the one presented here, then provide a reliable basis for further evolutionary genomic analyses when made available to the scientific community. The particular gene ortholog described here was characterized as part of a developing dataset to study the evolution of the Insulin/insulin-like growth factor signaling pathway (IIS) across the genus *Drosophila* . The Insulin/insulin-like growth factor signaling pathway (IIS) is a highly conserved signaling pathway in animals and is central to mediating organismal responses to nutrients (Hietakangas and Cohen 2009; Grewal 2009) . " *D* . *ananassae* (NCBI:txid7217) is part of the *melanogaster* species group within the subgenus *Sophophora* of the genus *Drosophila* (Sturtevant 1939; Bock and Wheeler 1972) . It was first described by Doleschall (1858). *D. ananassae* is circumtropical (Markow and O'Grady 2006; https://www.taxodros.uzh.ch, accessed 1 Feb 2023), and often associated with human settlement (Singh 2010) . It has been extensively studied as a model for its cytogenetic and genetic characteristics, and in experimental evolution (Kikkawa 1938; Singh and Yadav 2015) .” (Lawson et al, submitted).

We propose a gene model for the *D. ananassae* ortholog of the *D. melanogaster* Myc ( *Myc* ) gene. The genomic region of the ortholog corresponds to the uncharacterized protein LOC6502359 (RefSeq accession XP_044573530.1 ) in the dana_caf1 Genome Assembly of *D. ananassae* (GenBank Accession: GCA_000005115.1 ; Drosophila 12 Genomes Consortium 2007). This model is based on RNA-Seq data from *D. ananassae* ( SRP006203 , SRP007906 ; PRJNA257286 , PRJNA388952 - Graveley et al., 2011 *)* and *Myc* in *D. melanogaster* using FlyBase release FB2022_04 ( GCA_000001215.4 ; Larkin et al., 2021; Gramates et al., 2022; Jenkins et al., 2022).

*Myc* acts downstream of the insulin signaling pathway, with Myc protein accumulating in response to insulin through transcriptional and post-transcriptional mechanisms (Parisi et al., 2011) , resulting in the activation of genes involved in anabolic processes that promote cell growth (Terakawa et al., 2022) . *Myc* encodes a basic helix-loop-helix transcription factor in *Drosophila melanogaster* that is homologous to vertebrate *Myc* proto-oncogenes (Gallant et al., 1996) . In *Drosophila melanogaster* , *Myc* transcriptionally regulates a wide range of genes, including those that influence cell growth and metabolism (Teleman et al., 2008; Gallant 2013) .

***Synteny***

The reference gene, *Myc,* occurs on chromosome X in *D. melanogaster* and is flanked upstream by *new glue 4* ( *ng4* ), and *dunce* ( *dnc* ), which nests *Pre-intermoult gene 1* ( *Pig1* ), *salivary gland secretion 4* ( *Sgs4* ) and *CG10793* . *Myc* is flanked downstream by *CG12535* and *CG14269* . The *tblastn* search of *D. melanogaster* Myc-PB (query) against the *D. ananassae* (GenBank Accession: GCA_000005115.1 ) Genome Assembly (database) placed the putative ortholog of *Myc* within scaffold_12613 ( CH902663.1 ) at locus LOC6502359 (XP_ 044573530.1 )— with an E-value of 1e-42 and a percent identity of 36.30%. Furthermore, the putative ortholog is flanked upstream by LOC116655047 ( XP_032307915.1 ) and LOC6502358 ( XP_032307954.2 ) which nests LOC6502594 ( XP_032307960.1 ) and correspond to *ng4* , *dnc,* and *CG10793* in *D. melanogaster* (E-value: 4e-16, 0.0, and 0.0; identity:71.43%, 84.49% and 80.13%, respectively, as determined by *blastp* ; Figure 1A, A ltschul et al., 1990). The putative ortholog of *Myc* is flanked downstream by LOC6502362 ( XP_001967532.1 ) and LOC6502361 ( XP_001967530.1 ), which correspond to *CG12535* and *CG14269* in *D. melanogaster* (E-value: 7e-45 and 1e-104; identity: 47.80% and 83.16%, respectively, as determined by *blastp* ). The putative ortholog assignment for *Myc* in *D. ananassae* is supported by the following evidence: The genes surrounding the *Myc* ortholog are orthologous to the genes at the same locus in *D. melanogaster* and local synteny is nearly completely conserved, so we conclude that LOC6502359 is the correct ortholog of *Myc* in *D. ananassae* ( Figure 1A ).

***Protein Model***

*Myc* in *D. ananassae* has one unique protein-coding isoforms ( Figure 1B ), encoded by mRNA isoforms *Myc-RB* and *Myc-RA* that differ in their UTRs, and contain two CDSs. Relative to the ortholog in *D. melanogaster* , the RNA CDS number and protein isoform count is conserved. The sequence of Myc-PB in *D. ananassae* has 44.38% identity (E-value: 9e-77) with the protein-coding isoform Myc-PB in *D. melanogaster* , as determined by *blastp* ( Figure 1C ). Box W in red highlights tandem repeats of serine in CDS two, shown in Figure 1C *.* Coordinates of this curated gene model are stored by NCBI at GenBank/BankIt ( BK064666 and BK064667 ). These data are also archived in the CaltechDATA repository (see “Extended Data” section below).

***Special characteristics of the protein model***

**Regions of low conservation:** Lack of sequence similarity in CDSs one and two of *Myc-RA* and *Myc-RB* is displayed in the 28 *Drosophila* Species Conservation track ( Figure 1D ) within the vertical red boxes (X and Y). The most obvious lack of sequence similarity primarily exists at the start of the first CDS and in the middle of CDS two in many *Drosophila* species including *D. ananassae* which is indicated by the horizontal red box ( Figure 1D ). This lack of sequence similarity is likely due to the divergence of the species *D. ananassae* from *D. melanogaster.*

## Methods

Detailed methods including algorithms, database versions, and citations for the complete annotation process can be found in Rele et al. (2023). Briefly, students use the GEP instance of the UCSC Genome Browser v.435 (https://gander.wustl.edu ; Kent WJ et al., 2002; Navarro Gonzalez et al., 2021) to examine the genomic neighborhood of their reference IIS gene in the *D. melanogaster* genome assembly (Aug. 2014; BDGP Release 6 + ISO1 MT/dm6). Students then retrieve the protein sequence for the *D. melanogaster* target gene for a given isoform and run it using *tblastn* against their target *Drosophila* species genome assembly ( *D. ananassae* (GenBank Accession: GCA_000005115.1 ) on the NCBI BLAST server (https://blast.ncbi.nlm.nih.gov/Blast.cgi, Altschul et al., 1990) to identify potential orthologs. To validate the potential ortholog, students compare the local genomic neighborhood of their potential ortholog with the genomic neighborhood of their reference gene in *D. melanogaster* . This local synteny analysis includes at minimum the two upstream and downstream genes relative to their putative ortholog. They also explore other sets of genomic evidence using multiple alignment tracks in the Genome Browser, including BLAT alignments of RefSeq Genes, Spaln alignment of D. melanogaster proteins, multiple gene prediction tracks (e.g., GeMoMa, Geneid, Augustus), and modENCODE RNA-Seq from the target species. Genomic structure information (e.g., CDSs, CDS number and boundaries, number of isoforms) for the *D. melanogaster* reference gene is retrieved through the Gene Record Finder (https://gander.wustl.edu/~wilson/dmelgenerecord/index.html; Rele et al *.,* 2023). Approximate splice sites within the target gene are determined using *tblastn* using the CDSs from the *D. melanogaste* r reference gene. Coordinates of CDSs are then refined by examining aligned modENCODE RNA-Seq data, and by applying paradigms of molecular biology such as identifying canonical splice site sequences and ensuring the maintenance of an open reading frame across hypothesized splice sites. Students then confirm the biological validity of their target gene model using the Gene Model Checker (https://gander.wustl.edu/~wilson/dmelgenerecord/index.html; Rele et al., 2023), which compares the structure and translated sequence from their hypothesized target gene model against the *D. melanogaster* reference gene model. At least two independent models for each gene are generated by students under mentorship of their faculty course instructors. These models are then reconciled by a third independent researcher mentored by the project leaders to produce a final model like the one presented here. Note: comparison of 5' and 3' UTR sequence information is not included in this GEP CURE protocol.

## Extended Data

Description: GFF, FASTA, and PEP sequences for Myc in D ananassae. Resource Type: Dataset. DOI: 10.22002/0c391-eeh07

## References

[R1] Altschul SF, Gish W, Miller W, Myers EW, Lipman DJ (1990). Basic local alignment search tool.. J Mol Biol.

[R2] Bock IR, Wheeler MR. 1972. The Drosophila melanogaster species-group. The University of Texas Publication. 7213: 1-102.

[R3] Doleschall CL. 1858. Derde bijdrage tot de kennis der dipterologische fauna van Nederlandsch Indië. Natuurkundig tijdschrift voor Nederlandsch Indië. 17: 73.

[R4] Clark AG, Eisen MB, Smith DR, Bergman CM, Oliver B, Markow TA, Kaufman TC, Kellis M, Gelbart W, Iyer VN, Pollard DA, Sackton TB, Larracuente AM, Singh ND, Abad JP, Abt DN, Adryan B, Aguade M, Akashi H, Anderson WW, Aquadro CF, Ardell DH, Arguello R, Artieri CG, Barbash DA, Barker D, Barsanti P, Batterham P, Batzoglou S, Begun D, Bhutkar A, Blanco E, Bosak SA, Bradley RK, Brand AD, Brent MR, Brooks AN, Brown RH, Butlin RK, Caggese C, Calvi BR, Bernardo de Carvalho A, Caspi A, Castrezana S, Celniker SE, Chang JL, Chapple C, Chatterji S, Chinwalla A, Civetta A, Clifton SW, Comeron JM, Costello JC, Coyne JA, Daub J, David RG, Delcher AL, Delehaunty K, Do CB, Ebling H, Edwards K, Eickbush T, Evans JD, Filipski A, Findeiss S, Freyhult E, Fulton L, Fulton R, Garcia AC, Gardiner A, Garfield DA, Garvin BE, Gibson G, Gilbert D, Gnerre S, Godfrey J, Good R, Gotea V, Gravely B, Greenberg AJ, Griffiths-Jones S, Gross S, Guigo R, Gustafson EA, Haerty W, Hahn MW, Halligan DL, Halpern AL, Halter GM, Han MV, Heger A, Hillier L, Hinrichs AS, Holmes I, Hoskins RA, Hubisz MJ, Hultmark D, Huntley MA, Jaffe DB, Jagadeeshan S, Jeck WR, Johnson J, Jones CD, Jordan WC, Karpen GH, Kataoka E, Keightley PD, Kheradpour P, Kirkness EF, Koerich LB, Kristiansen K, Kudrna D, Kulathinal RJ, Kumar S, Kwok R, Lander E, Langley CH, Lapoint R, Lazzaro BP, Lee SJ, Levesque L, Li R, Lin CF, Lin MF, Lindblad-Toh K, Llopart A, Long M, Low L, Lozovsky E, Lu J, Luo M, Machado CA, Makalowski W, Marzo M, Matsuda M, Matzkin L, McAllister B, McBride CS, McKernan B, McKernan K, Mendez-Lago M, Minx P, Mollenhauer MU, Montooth K, Mount SM, Mu X, Myers E, Negre B, Newfeld S, Nielsen R, Noor MA, O'Grady P, Pachter L, Papaceit M, Parisi MJ, Parisi M, Parts L, Pedersen JS, Pesole G, Phillippy AM, Ponting CP, Pop M, Porcelli D, Powell JR, Prohaska S, Pruitt K, Puig M, Quesneville H, Ram KR, Rand D, Rasmussen MD, Reed LK, Reenan R, Reily A, Remington KA, Rieger TT, Ritchie MG, Robin C, Rogers YH, Rohde C, Rozas J, Rubenfield MJ, Ruiz A, Russo S, Salzberg SL, Sanchez-Gracia A, Saranga DJ, Sato H, Schaeffer SW, Schatz MC, Schlenke T, Schwartz R, Segarra C, Singh RS, Sirot L, Sirota M, Sisneros NB, Smith CD, Smith TF, Spieth J, Stage DE, Stark A, Stephan W, Strausberg RL, Strempel S, Sturgill D, Sutton G, Sutton GG, Tao W, Teichmann S, Tobari YN, Tomimura Y, Tsolas JM, Valente VL, Venter E, Venter JC, Vicario S, Vieira FG, Vilella AJ, Villasante A, Walenz B, Wang J, Wasserman M, Watts T, Wilson D, Wilson RK, Wing RA, Wolfner MF, Wong A, Wong GK, Wu CI, Wu G, Yamamoto D, Yang HP, Yang SP, Yorke JA, Yoshida K, Zdobnov E, Zhang P, Zhang Y, Zimin AV, Baldwin J, Abdouelleil A, Abdulkadir J, Abebe A, Abera B, Abreu J, Acer SC, Aftuck L, Alexander A, An P, Anderson E, Anderson S, Arachi H, Azer M, Bachantsang P, Barry A, Bayul T, Berlin A, Bessette D, Bloom T, Blye J, Boguslavskiy L, Bonnet C, Boukhgalter B, Bourzgui I, Brown A, Cahill P, Channer S, Cheshatsang Y, Chuda L, Citroen M, Collymore A, Cooke P, Costello M, D'Aco K, Daza R, De Haan G, DeGray S, DeMaso C, Dhargay N, Dooley K, Dooley E, Doricent M, Dorje P, Dorjee K, Dupes A, Elong R, Falk J, Farina A, Faro S, Ferguson D, Fisher S, Foley CD, Franke A, Friedrich D, Gadbois L, Gearin G, Gearin CR, Giannoukos G, Goode T, Graham J, Grandbois E, Grewal S, Gyaltsen K, Hafez N, Hagos B, Hall J, Henson C, Hollinger A, Honan T, Huard MD, Hughes L, Hurhula B, Husby ME, Kamat A, Kanga B, Kashin S, Khazanovich D, Kisner P, Lance K, Lara M, Lee W, Lennon N, Letendre F, LeVine R, Lipovsky A, Liu X, Liu J, Liu S, Lokyitsang T, Lokyitsang Y, Lubonja R, Lui A, MacDonald P, Magnisalis V, Maru K, Matthews C, McCusker W, McDonough S, Mehta T, Meldrim J, Meneus L, Mihai O, Mihalev A, Mihova T, Mittelman R, Mlenga V, Montmayeur A, Mulrain L, Navidi A, Naylor J, Negash T, Nguyen T, Nguyen N, Nicol R, Norbu C, Norbu N, Novod N, O'Neill B, Osman S, Markiewicz E, Oyono OL, Patti C, Phunkhang P, Pierre F, Priest M, Raghuraman S, Rege F, Reyes R, Rise C, Rogov P, Ross K, Ryan E, Settipalli S, Shea T, Sherpa N, Shi L, Shih D, Sparrow T, Spaulding J, Stalker J, Stange-Thomann N, Stavropoulos S, Stone C, Strader C, Tesfaye S, Thomson T, Thoulutsang Y, Thoulutsang D, Topham K, Topping I, Tsamla T, Vassiliev H, Vo A, Wangchuk T, Wangdi T, Weiand M, Wilkinson J, Wilson A, Yadav S, Young G, Yu Q, Zembek L, Zhong D, Zimmer A, Zwirko Z, Jaffe DB, Alvarez P, Brockman W, Butler J, Chin C, Gnerre S, Grabherr M, Kleber M, Mauceli E, MacCallum I, Drosophila 12 Genomes Consortium. (2007). Evolution of genes and genomes on the Drosophila phylogeny.. Nature.

[R5] Gallant P (2013). Myc function in Drosophila.. Cold Spring Harb Perspect Med.

[R6] Gallant P, Shiio Y, Cheng PF, Parkhurst SM, Eisenman RN (1996). Myc and Max homologs in Drosophila.. Science.

[R7] Gramates LS, Agapite J, Attrill H, Calvi BR, Crosby MA, Dos Santos G, Goodman JL, Goutte-Gattat D, Jenkins VK, Kaufman T, Larkin A, Matthews BB, Millburn G, Strelets VB, the FlyBase Consortium. (2022). Fly Base: a guided tour of highlighted features.. Genetics.

[R8] Grewal SS (2008). Insulin/TOR signaling in growth and homeostasis: a view from the fly world.. Int J Biochem Cell Biol.

[R9] Hietakangas V, Cohen SM (2009). Regulation of tissue growth through nutrient sensing.. Annu Rev Genet.

[R10] Jenkins VK, Larkin A, Thurmond J, FlyBase Consortium (2022). Using FlyBase: A Database of Drosophila Genes and Genetics.. Methods Mol Biol.

[R11] Kent WJ, Sugnet CW, Furey TS, Roskin KM, Pringle TH, Zahler AM, Haussler D (2002). The human genome browser at UCSC.. Genome Res.

[R12] Kikkawa Hideo (1938). Studies on the genetics and cytology ofDrosophila ananassae. Genetica.

[R13] Larkin A, Marygold SJ, Antonazzo G, Attrill H, Dos Santos G, Garapati PV, Goodman JL, Gramates LS, Millburn G, Strelets VB, Tabone CJ, Thurmond J, FlyBase Consortium. (2021). FlyBase: updates to the Drosophila melanogaster knowledge base.. Nucleic Acids Res.

[R14] Lawson ME, Mcabee M, Lucas RA, Tanner S, Wittke-Thompson J, Pelletier TA, et al., Rele, CP. 2024. Gene model for the ortholog of Ilp5 in Drosophila ananassae (submitted). 10.17912/micropub.biology.000782 PMC11664428 39717145

[R15] Markow TA and O’Grady P. 2005. Drosophila: A guide to species identification and use. London: Academic Press. ISBN: 978-0-12-473052-6

[R16] Mudge JM, Harrow J (2016). The state of play in higher eukaryote gene annotation.. Nat Rev Genet.

[R17] Navarro Gonzalez J, Zweig AS, Speir ML, Schmelter D, Rosenbloom KR, Raney BJ, Powell CC, Nassar LR, Maulding ND, Lee CM, Lee BT, Hinrichs AS, Fyfe AC, Fernandes JD, Diekhans M, Clawson H, Casper J, Benet-Pagès A, Barber GP, Haussler D, Kuhn RM, Haeussler M, Kent WJ (2021). The UCSC Genome Browser database: 2021 update.. Nucleic Acids Res.

[R18] Parisi F, Riccardo S, Daniel M, Saqcena M, Kundu N, Pession A, Grifoni D, Stocker H, Tabak E, Bellosta P (2011). Drosophila insulin and target of rapamycin (TOR) pathways regulate GSK3 beta activity to control Myc stability and determine Myc expression in vivo.. BMC Biol.

[R19] Raney BJ, Dreszer TR, Barber GP, Clawson H, Fujita PA, Wang T, Nguyen N, Paten B, Zweig AS, Karolchik D, Kent WJ (2013). Track data hubs enable visualization of user-defined genome-wide annotations on the UCSC Genome Browser.. Bioinformatics.

[R20] Rele Chinmay P., Sandlin Katie M., Leung Wilson, Reed Laura K. (2023). Manual annotation of Drosophila genes: a Genomics Education Partnership protocol. F1000Research.

[R21] Singh BN (2010). Drosophila ananassae: a good model species for genetical, behavioural and evolutionary studies.. Indian J Exp Biol.

[R22] Singh BN, Yadav JP (2015). Status of research on Drosophila ananassae at global level.. J Genet.

[R23] Sturtevant AH (1939). On the Subdivision of the Genus Drosophila.. Proc Natl Acad Sci U S A.

[R24] Teleman AA, Hietakangas V, Sayadian AC, Cohen SM (2008). Nutritional control of protein biosynthetic capacity by insulin via Myc in Drosophila.. Cell Metab.

[R25] Tello-Ruiz MK, Marco CF, Hsu FM, Khangura RS, Qiao P, Sapkota S, Stitzer MC, Wasikowski R, Wu H, Zhan J, Chougule K, Barone LC, Ghiban C, Muna D, Olson AC, Wang L, Ware D, Micklos DA (2019). Double triage to identify poorly annotated genes in maize: The missing link in community curation.. PLoS One.

[R26] Terakawa A, Hu Y, Kokaji T, Yugi K, Morita K, Ohno S, Pan Y, Bai Y, Parkhitko AA, Ni X, Asara JM, Bulyk ML, Perrimon N, Kuroda S (2022). Trans-omics analysis of insulin action reveals a cell growth subnetwork which co-regulates anabolic processes.. iScience.

